# Phytochemicals as Potential Anticancer Drugs: Time to Ponder Nature's Bounty

**DOI:** 10.1155/2020/8602879

**Published:** 2020-01-31

**Authors:** Mohammad Arif Ashraf

**Affiliations:** Department of Biology, University of Massachusetts, Amherst 01003, MA, USA

## Abstract

Medicinal plants have been used from the beginning of human civilization, which is mostly evident from the ancient script and traditional herbal medicine recipe. Despite the historically enriched demonstration about the use of plant as therapeutics, the pharmaceutical industries lack interest on phytochemical research compared with synthetic drug. Mostly, the absence of information about plant-based medicinal therapeutics is responsible to draw the attention of researchers to think about natural products as potential drug for detrimental diseases, such as cancer. This review will cover about clinically successful plant-based anticancer drugs and underappreciated, but potential, drugs to bridge the information gap between plant biologists and clinical researchers. Additionally, unprecedented advancement of synthetic chemistry, omics study to pin point the target genes/proteins, and efficient drug delivery system have made it easier for researchers to develop a phytochemical as an efficient anticancer drug.

## 1. Introduction

Undoubtedly, we are living in a time when cancer is epidemic and one of the medical challenges of this century. Statistically, it is a devastating number to deal. According to the Cancer Research UK, 17 million new cases had been reported, and among them, 9.6 million patients ended up to death in 2018. If it continues in such a rate, by 2040, there will be 27.5 million new cancer patient each year. At the same time, if we wonder, how far we came to cure cancer—forget about that—because our understanding about cancer is like looking at the White House from the international space station, where we certainly do not realize the color or the power of the stuff we are looking at. However, if we take a step back before meddling our mind with thousands of target genes, proteins, and possible or probable drugs, the fundamental idea of different types of cancer is “uncontrolled cell division” [1]. In short, cell division is a rudimentary process from the very beginning of the existence of life in the universe. Symmetric cell division leads to proliferation and asymmetric cell division is an informative step for differentiation. It has been reported that uncontrolled symmetric cell division is the major factor for causing cancer [2]. It is quite reasonable to aim anticancer drugs to control the cell cycle machineries [3]. In recent years, along with the effort of traditional anticancer drug discovery approaches, which is time-consuming and expensive, there is a search for anticancer drug from plant-derived bioactive compounds. Furthermore, drug discovery and development from plant-based compounds are also inexpensive compared with conventional synthetic compounds [4].

The plants are visibly an efficient provider of food and shelter, but the role of plants as a source of medicine is underappreciated. Human civilization is using plant as a source of food, shelter, and medicine for almost same time [5]. The contribution of plant as medicine was neglected due to the lack of precise biochemical and pharmacological mechanisms. Surprisingly, in nature, plants are continuously and extensively exposed to natural pollutants, carcinogens, and toxic metals [6–9] compared with human. Apart from the classic crown gall disease [10], the example of uncontrolled cell division in plant is insignificant compared with the animal system. At the same time, plants synthesize divergent of secondary metabolites, mostly used for their defense and response to environmental cues, such as biotic and abiotic stresses [11–14]. In most of the cases, we have limited idea how plants tightly regulate their cell cycle machineries endogenously even after enormous exposure to hazardous components. Till date, several plant-derived compounds such as taxol [15], vinblastine [16], topotecan [17], and many more have been used as anticancer drugs successfully in clinical studies.

In this review, the existing successful plant-based anticancer drugs will be explored first and then the future direction of this emerging area and how the advancement of drug delivery system and cell type-specific production of anticancer drugs will uplift plant-based compounds as anticancer agents will be discussed.

## 2. Plant-Based Cancer Treatment

### 2.1. Looking Back to the Past to Find the Future

The most ancient text described the use of plant material as medicine was found in the Sumerian clay slab from Nagpur, India, and written approximately 5000 years ago. It contains information about using recent days' popular poppy, henbane, and mandrake as therapeutics [18]. Next, oldest evidence of medicinal plant was demonstrated in ancient Chinese literature written by Emperor Shen Nung circa 2500 BC [19, 20]. In a continuous historical effort, Theophrastus, known as “the father of botany,” first established the botanical science, documented in his book “*De Causis Plantarum*,” and classified several hundred medicinal plants [21, 22]. In addition, historically prominent Greek physician, pharmacologist, and botanist Pedanius Dioscorides wrote a 5-volume book, “*De Materia Medica*,” on the medicinal use of plant [23]. His book and research were enormously successful, and he was hired by Roman army as a physician. The legacy of Roman Empire on medicinal plant study was further carried by Muslim scholars during the Islamic Golden Age. For instance, Islamic scholar Ibn Baitar described more than thousand medicinal plants in his book, “*Liber Magnae Collectionis Simplicum Alimentorum Et Medicamentorum*” [24]. The knowledge of medicinal plants from ancient literature and text came to light through Carl Linnaeus's classification system, described in his book “*Species Plantarum*” [25].

In the early 19^th^ century, the advent of advanced synthetic chemistry helps us to decipher the mechanism, isolation and synthesis of active compounds from popular medicinal plants, such as poppy, ipecacuanha, strychnos, quinine, and pomegranate [26]. Despite the enriched history and success of medicinal plants, during the late 19^th^ and early 20^th^ centuries, the research on medicinal plant did not progress as it was supposed to. The reluctance of pharmaceutical industries about plant-based components caused a significant shift of focus from plant to synthetic chemistry on drug development [27].

Fortunately, the gear has been shifted in recent years. In 2015, the Nobel Prize in physiology and medicine was awarded to Tu Youyou for her discovery of artemisinin and dihydroartemisinin as antimalarial drug and highlight of the importance of plant-based components as a potentially powerful source of drug discovery. During the Vietnam War, Ho Chi Minh urged to develop antimalarial drugs for his soldiers. Tu Youyou became the part of that project to find the antimalarial treatment, and she screened over 2,000 traditional herbal medicines and discovered *Artemisia annua* recipe [28].

The success story of antimalarial drug based on traditional herbal medicine is not an isolated story; rather, it is a tiny part of plant-based arsenal as potential drugs. As a result, the significant effort on finding therapeutic agents for cancer treatment has been focused on plant-based compounds by the National Cancer Institute (NCI), USA. NCI-initiated Cancer Moonshot^SM^ project, aimed to accelerate the cancer research by making more cancer therapeutics available for patients, is focused on phytochemicals. As a part of this project, they have established a repertoire of natural products, and their purified chemical components to make them available for researchers to find new anticancer drugs.

Till date, several plant-based compounds have been reported for their anticancer activity, and among them, a good number of compounds is clinically successful as well. I have tried to summarize some important phytochemicals as potential anticancer drugs in Table 1 and Section 2.2.

### 2.2. Success Stories of Medicinal Plant-Based Anticancer Drug

Till date, more than thousand plants species have been identified with noteworthy anticancer potential [90, 91]. The isolation of the vinca alkaloids, vinblastine [92] from the Madagascar periwinkle, and *Catharanthus roseus* G. Don. (Apocynaceae) is one of the major examples of anticancer medication. This along with vincristine and other cancer chemotherapeutic drugs are used for the treatment of a range of cancers such as leukemias, lymphomas, advanced testicular cancer, breast and lung cancers, and Kaposi's sarcoma [30, 91]. The discovery of paclitaxel (Taxol) [93] from the bark of the Pacific Yew, *Taxus brevifolia* Nutt. (Taxaceae), is another major success story in natural product drug discovery. Utilization of various parts of *Taxus brevifolia* from which paclitaxel was discovered and other *Taxus* species (e.g., *Taxus Canadensis* Marshall and *Taxus baccata* L.) by several Native American Tribes kindle the idea of indigenous knowledge-based medicinal plants [30, 91]. Another potent plant-acquired active compound, Homoharringtonine [94], was extracted from the Chinese tree *Cephalotaxus harringtonia* var. drupacea (Sieb and Zucc.) (Cephalotaxaceae) and has been used successfully for a long time in China in a racemic mixture with harringtonine for the treatment of acute myelogenous leukemia [30]. Elliptinium, a derivative of ellipticine, isolated from a Fijian medicinal plant *Bleekeria vitensis* A. C. Sm., is shipped to France for the treatment of breast cancer [30, 91]. These events represent only the surface of the success story of plant-based anticancer drug discovery with a promise to find more in the near future [91].

### 2.3. Toddler's Step Leads to the Unknown Horizon

**Figure 1:**
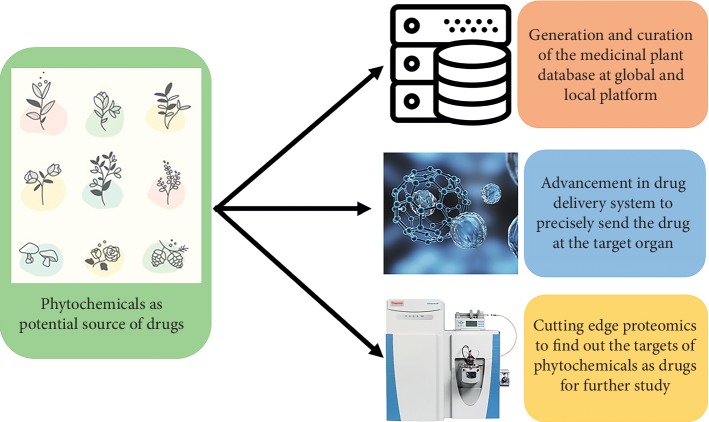
Phytochemicals can be used as drugs based on medicinal plant database, omics study to find the target, and efficient drug delivery system.

The question is whether long-term ignorance of natural products for drug development can fit into the multibillion-dollar pharmaceutical industry? The answer certainly depends on multiple factors. However, the bright side is that the recent advancement of multiple medicinal plant database from local and global researchers, cutting edge omics technique to accelerate to find the drug targets and unprecedented improvement on drug delivery system (Figure 1).

Over the last decade, along with the effort of Moonshot^SM^ project, local scientists from different parts of the work tried to curate the information of traditional herbal practice, preparation, recipe, and their ailments [12, 95–97]. Furthermore, to find out the target of potential phytochemicals is easier than ever, because of advancement of genomics, proteomics, transcriptomics, and metabolomics in recent years [91, 98–100]. The final frontier of using natural products as cancer treatment depends on the efficient drug delivery system. Fortunately, the advent of nanotechnology for drug delivery system has fast-forwarded this sector over the last few years [101, 102].

## 3. Conclusions

If we assume the research avenue of phytochemicals as potential cancer therapeutics as an image of a pyramid, this review has demonstrated a piece of stone from that pyramid. However, the idea of pushing natural products' research on drug discovery and development requires constant update and well-documented literature. This review paper will take the reader from the ancient history of herbal medicinal practice to the modern day's isolation, purification, identification, biosynthesis, in vitro or in vivo study, drug development, efficient delivery of drugs, and therapeutic trial.

## Figures and Tables

**Table 1:** Known phytochemicals, their source, and therapeutic use.

Phytochemical	Source	Therapeutic use	Reference
5-Fluorouracil	*Withania somnifera*	Human cervical cancer cell	[29]
Vindesine	*Catharanthus roseus*	Leukemias, testicular, breast and lung cancer	[30]
Vincristine	*Catharanthus roseus*	Lymphocytic leukemia	[30]
Vinblastine	*Catharanthus roseus*	Lymphocytic leukemia	[30]
Colchicine	*Colchicum autumnale*	Multiple solid tumors	[31]
Larotaxel	*Taxus baccata*	Breast, bladder, and pancreatic cancer	[32]
Cabazitaxel	*Taxus baccata*	Prostate cancer	[33]
Paclitaxel	*Taxus brevifolia*	Breast and ovarian cancer	[30]
Bullatacin	*Annona squamosa*	Liver cancer	[34]
Bryophyllin A	*Bryophyllum pinnatum*	Cervical cancer	[35]
Harmine	*Peganum harmala*	Breast cancer	[36]
Artemisinin	*Artemisia annua*	Liver, breast, and pancreatic cancer	[37]
Tannins	*Debregeasia saeneb*	Internal tumors	[38]
Theabrownin	*Camellia sinensis*	Lung cancer	[39]
Solamargine	*Solanum nigrum*	Breast, liver, lung, and skin cancer	[40]
Psoralidin	*Psoralea corylifolia*	Stomach and prostate cancer	[41]
Xanthatin	*Xanthium strumarium*	Lymphocytic leukemia and liver cancer	[42]
Thymoquinone	*Nigella sativa*	Colon, prostate, breast, and pancreas cancer	[43]
Kaempferol galactoside	*Bauhinia variegata*	Breast, lung, and liver cancer	[43]
Withaferin A, D	*Withania somnifera*	Breast, cervix, prostate, and colon cancer	[44]
Ginger	*Zingiber officinale*	Ovary, cervix, colon, liver, and urinary caner	[45]
Silibinin	*Sylibum marianum*	Lung, liver, skin, colon, and prostate cancer	[46]
Luteolin	*Capsicum annuum*	Colorectal cancer	[47]
Colchicine	*Colchicum autumnale*	Hodgkin's lymphoma, chronic granulocytic leukemia	[48]
Skimmianine	*Aegle marmelos*	Liver cancer	[49]
Boswellic acid	*Boswellia serrata*	Prostate cancer	[50]
Silymarin	*Sylibum marianum*	Colorectal cancer and colon cancer	[51]
Curcumin	*Curcuma longa*	Colon adenocarcinoma	[52]
Podophyllotoxin	*Podophyllum peltatum*	Non-small-cell lung carcinoma	[53]
Andrographolide	*Andrographis paniculata*	Colon cancer	[47]
Podophyllotoxin	*Podophyllum hexandrum*	Breast, ovary, lung, liver, bladder, and testis cancer	[54]
Betulinic acid	*Betula utilis*	Melanomas	[55]
Panaxadiol	*Panax ginseng*	Human colon cancer	[56]
Gossypol	*Gossypium hirsutum*	Colorectal cancer	[57]
Chrysin	*Passiflora caerulea*	Colorectal cancer	[58]
Plumbagin	*Plumbago zeylanica*	Liver, fibrosarcoma, leukemia, and breast cancer	[59]
6-Shogaol	*Zingiber officinale*	Ovary cancer	[60]
Curcumin	*Curcuma longa*	Breast, lung, colon, prostate esophagus, liver, and skin cancer	[61]
Ursolic acid	*Oldenlandia diffusa*	Lungs, ovary, uterus, stomach, liver, colon, rectum, and brain cancer	[62]
Isoliquiritigenin	*Glycyrrhiza uralensis*	Human lung cancer	[63]
Punarnavine	*Boerrhavia diffusa*	Malignant melanoma cancer	[64]
Procyanidins	*Vitis vinifera*	Human colon cancer	[65]
Resveratrol	*Polygonum cuspidatum*	Colorectal, skin, and liver cancer	[66]
Damnacanthal	*Morinda citrifolia*	Lung cancer, sarcomas	[67]
Gossypol	*Gossypium hirsutum*	Breast, stomach, liver, prostate, and bladder cancer	[68]
Niazinine A	*Moringa oliefera*	Blood cancer	[69]
Amooranin	*Amoora rohituka*	Lymphocytic leukemia	[70]
Betulinic acid	*Ziziphus rugosa*	Cytotoxicity against human melanoma cells	[71]
Asiatic acid	*Centella asiatica*	Melanoma, glioblastoma, breast cancer	[72]
Gallic acid	*Leea indica*	Ehrlich ascites carcinoma	[73]
Combretastatins	*Combretum caffrum*	Colon, leukemia, and lung cancer	[74]
Lycopene	*Solanum lycopersicum*	Prostate and colon cancer	[75]
Plumbagin	*Plumbago zeylanica*	Blood and skin cancer	[76]
Cannabinoid	*Cannabis sativa*	Lung, pancreas, breast, prostate, and colorectal cancer	[77]
Silymarin	*Sylibum marianum*	Colorectal cancer	[78]
Tylophorine	*Tylophora indica*	Breast cancer	[74]
Saffron	*Saffron crocus*	Liver, lung cancer and pancreatic cancer	[79]
nab-paclitaxel	*Taxus brevifolia*	Ovarian and breast cancer	[80]
Cyanidin	*Vitis vinifera*	Colon cancer	[81]
Actein	*Actaea racemosa*	Liver and breast cancer	[82]
Betulinic acid	*Betula Sp.*	Human melanoma xenografts and leukemia	[30]
Allin	*Allium sativum*	Carcinoma of human mammary gland	[83]
Neferine	*Nelumbo nucifera*	Liver cancer	[84]
Calcaelin	*Calvatia caelata*	Breast and spleen cancer cells	[85]
Lentinan	*Lentinus edodes*	Sarcoma-180 in mice	[86]
Schizophyllan	*Schizophyllum commune*	Head and neck cancer	[87]
Apigenin	*Matricaria chamomilla*	Colorectal cancer	[88]
Vitex	*Vitex agnus-castu*	Human uterine, ovarian, cervical, and breast cancer	[89]
